# Supplementation with L-Glutamine and L-Alanyl-L-Glutamine Changes Biochemical Parameters and Jejunum Morphophysiology in Type 1 Diabetic Wistar Rats

**DOI:** 10.1371/journal.pone.0143005

**Published:** 2015-12-14

**Authors:** Carlos Vinicius D. da Rosa, Silvia C. S. F. Azevedo, Roberto B. Bazotte, Rosane M. Peralta, Nilza C. Buttow, Maria Montserrat D. Pedrosa, Vilma A. F. de Godoi, Maria Raquel M. Natali

**Affiliations:** 1 Department of Morphological Sciences, State University of Maringá, Maringá, Paraná, Brazil; 2 Department of Physiological Sciences, State University of Maringá, Maringá, Paraná, Brazil; 3 Department of Pharmacology and Therapeutics, State University of Maringá, Maringá, Paraná, Brazil; 4 Department of Biochemistry, State University of Maringá, Maringá, Paraná, Brazil; University of North Carolina at Chapel Hill, UNITED STATES

## Abstract

We evaluated the effects of the supplementation with L-glutamine and glutamine dipeptide (GDP) on biochemical and morphophysiological parameters in streptozotocin-diabetic rats. For this purpose, thirty animals were distributed into six groups treated orally (gavage) during thirty days: non diabetic rats (Control) + saline, diabetic + saline; Control + L-glutamine (248 mg/kg), Diabetic + L-glutamine (248 mg/kg), Control + GDP (400 mg/kg), Diabetic + GDP (400 mg/kg). Diabetes was induced by an intravenous injection of streptozotocin (60 mg/kg) and confirmed by fasting glucose ≥ 200 mg/dL. Physiological parameters, i.e., body mass, food intake, blood glucose, water intake, urine and faeces were evaluated during supplementation. After the period of supplementation, the animals were euthanized. The blood was collected for biochemical assays (fructosamine, transaminases, lipid profile, total protein, urea, ammonia). Moreover, the jejunum was excised and stored for morphophysiological assays (intestinal enzyme activity, intestinal wall morphology, crypt proliferative index, number of serotoninergic cells from the mucosa, and vipergic neurons from the submucosal tunica). The physiological parameters, protein metabolism and intestinal enzyme activity did not change with the supplementation with L-glutamine or GDP. In diabetic animals, transaminases and fructosamine improved with L-glutamine and GDP supplementations, while the lipid profile improved with L-glutamine. Furthermore, both forms of supplementation promoted changes in jejunal tunicas and wall morphometry of control and diabetic groups, but only L-glutamine promoted maintenance of serotoninergic cells and vipergic neurons populations. On the other hand, control animals showed changes that may indicate negative effects of L-glutamine. Thus, the supplementation with L-glutamine was more efficient for maintaining intestinal morphophysiology and the supplementation with GDP was more efficient to the organism as a whole. Thus, we can conclude that local differences in absorption and metabolism could explain the differences between the supplementation with L-glutamine or GDP.

## Introduction

L-glutamine is considered a conditionally essential amino acid [1]. It is the precursor of peptides, proteins, neurotransmitters, nitrogenous bases and is used as an energy source by various organs such as the intestine. Other functions are assigned to this amino acid, such as maintenance of cell proliferation, immune function, acid-base balance and regulation of gene expression [2].

However, there are limitations of using L-glutamine as a supplement, such as low solubility in water and instability, especially during heat sterilization and prolonged storage. This led to the development of more stable synthetic forms, such as dipeptides with L-glutamine residues that are highly soluble in water and more resistant to thermal shock and prolonged storage [3].

In humans, approximately half of the oral L-glutamine is used by the enterocytes, generating a plasma increase around 50% of the amount supplied [4]. However, the intestinal absorption of L-glutamine in the form of glutamine dipeptide (GDP) is more effective than the free form [5].

Diabetes is a chronic disease characterized by decreased blood levels of L-glutamine [6]. Diabetes also affects the gastrointestinal tract, generating enzymatic, morphological and functional changes [7–9]. The hyperglycemia in human diabetes increases oxidative stress [10], which affects the enteric nervous system (ENS), composed of the submucous and myenteric ganglionated plexuses, which coordinate secretion, blood flow and motility of the digestive tract [9].

It is suggested that in diabetes the supplementation with L-glutamine can positively act reducing the oxidative stress and enteric neurodegeneration [10–13]. Moreover, considering that the intestinal absorption of peptides is increased in diabetes [7], supplementation with L-glutamine or GDP could influence the tissue response.

Thus, the purpose of this study was to evaluate the effects of the supplementation with L-glutamine or GDP on biochemical and morphophysiological parameters in streptozotocin-diabetic rats.

## Materials and Methods

### Drugs and chemicals

L-glutamine and GDP were obtained from Ajinomoto, Japan. Streptozotocin was purchased from Sigma-Aldrich, USA; glucometer and strips Optium Xceed were from Abbott, Brazil; while Thionembutal was purchased from Abbott Laboratories, USA. The blood laboratory kits were obtained from Gold Analisa Diagnostics Ltd., Brazil.

Anti-PCNA, anti-serotonin, secondary Alexa Fluor 488 antibodies, and Prolong Gold Antifade were purchased from Life Technologies, USA. Anti-VIP antibody was obtained from Bachem Americas, EUA. All other reagents were of the best quality available.

### Animals and treatment

Thirty male Wistar rats (*Rattus norvegicus*, 50 days, 188,9±9,4 g), from the Central Animal House of the State University of Maringá, were kept under controlled temperature (23°C / 25°C) and 12 h light/dark cycles, receiving standard diet (Nuvital®—Nuvilab, Colombo, PR, Brazil) and water *ad libitum*. All experimental protocols were approved by the Ethics Commission on the Use of Animals (CEUA/UEM—137/2013).

The animals were distributed into 6 groups (n = 5) that received by gavage during 30 days: control group (C group) and diabetic group (D group) received saline; control glutamine group (CG group) and diabetic glutamine group (DG group) received L-glutamine (248 mg/kg); control GDP group (CGDP group) and diabetic GDP group (DGDP group) received GDP (400 mg/kg). During the 30 days of the experimental period, body mass, food intake, water ingestion, urine volume, blood glucose and faeces were evaluated.

### Induction and confirmation of the diabetic state

The animals in groups D, DG and DGDP, after overnight fasting (14 h), received an intravenous injection of streptozotocin (60 mg/kg body mass) dissolved in citrate buffer, pH 4.5 (10 mM), to induce experimental type 1 diabetes. Animals with glycemia above 200 mg/dL (glucometer and strips) were considered diabetic [14]. The control animals (C, CG and CGDP) received an intravenous injection of citrate buffer.

### Tissue collection and material processing

After thirty days of supplementation, the animals were intraperitoneally anesthetized (40 mg/kg body mass) with intraperitoneal thionembutal. Blood samples were collected by cardiac puncture and the serum was used to measure total protein, triglycerides, total cholesterol and fractions, aspartate aminotransferase (AST), alanine aminotransferase (ALT); fructosamine; ammonia and urea.

Vertical laparotomy was made for collection and measurement of the length of the small intestine (SI). Subsequently, jejunal samples were separated and washed in phosphate buffered saline (PBS, 0.1 M, pH 7.3), and fixed either (1) in liquid nitrogen for analysis of the specific activity of intestinal enzymes; or (2) in 4% paraformaldehyde for histological and immunohistochemical processing.

### Analysis of intestinal enzymes

Samples of the jejunum were macerated, suspended in sodium phosphate buffer (50 mM, pH 6.5) and centrifuged under refrigeration. The supernatant was used to determine the levels of alkaline phosphatase [15], lipase [16], β-galactosidase [15] and maltase [17] in a spectrophotometer (Shimadzu UV-VIS—UV1800).

A unit of enzyme activity (U) was defined as the amount of enzyme that produced 1.0 μmol per mL of product per min under the assay conditions. The specific activity was expressed as U/g of jejunum (wet weight).

### Processing and histological analysis

After 6 h of fixation in 4% paraformaldehyde, jejunum samples were dehydrated in increasing series of alcohols, cleared in xylene and embedded in paraffin. Then, 6 **μ**m thick semiserial transversal sections were made with Leica RM 2145 microtome.

#### Morphometric analysis of the intestinal wall components

The histological sections were stained with hematoxylin and eosin (HE) for morphologic and morphometric analysis of jejunal wall components. The images were captured under a light microscope (Olympus BX41, Olympus America Inc., New York, USA) coupled to a high resolution camera (Olympus Q Color 3 Olympus America Inc., New York, USA) under a 10x objective to analyze the total wall thickness, mucosa and submucosa, and height of the villi. The crypt depth was measured under a 20x objective. One hundred measurements of each parameter were performed (10 points per section) per animal using the program Image Pro Plus 4.5 (Media Cybernetics, Maryland, USA).

#### Proliferating cells and serotoninergic indexes

Histological sections of the jejunum kept on slides with poly-L-lysine were deparaffinized, hydrated and treated with H_2_O_2_ (3%) in methanol for 10 min to eliminate endogenous peroxidase activity. After two 5 min washes with phosphate-buffered saline (PBS; pH 7.4; 0.1 M), the sections were blocked with a solution containing goat serum (10%) for 10 min. The tissues were then incubated with a solution containing anti-PCNA (proliferating cell nuclear antigen) (Cat# 18–0110, AB_86659) or rabbit anti-serotonin primary antibody (Cat# 18–0077, AB_86641) diluted 1:200 in PBS for 2 h. After two 5 min washes with PBS (pH 7.4; 0.1 M), the sections were incubated with biotinylated secondary antibody (broad spectrum kit solution) for 10 min, washed again, and treated with the streptavidin-peroxidase conjugate for 10 min. After further washes with PBS to remove excess enzyme conjugate, the immunohistochemical reaction was revealed by diaminobenzidine (DAB) in PBS and H_2_O_2_ for 15 min. After washing in distilled water, the sections were counterstained with hematoxylin, dehydrated in ethanol, diaphanized in xylene, and mounted under coverslips with Permount synthetic resin. All the procedures were performed at room temperature. The Histostain Plus Kit was used to perform this technique

To assess cell proliferation (PCNA), the slides were analyzed under light microscope, under 40x objective; 2500 crypt cells per animal were recorded to obtain the percentage of labeled to unlabeled cells (proliferation index). In the immunostained serotoninergic cells 2500 epithelial cells from longitudinally oriented villi and crypts were counted, to obtain the ratio of labeled cells. For both techniques, the index was calculated by the number of labeled cells*100 / total number of cells counted.

### Labeling and quantification of immunoreactive VIP-neurons

After fixation in paraformaldehyde, jejunal samples were dissected under a stereomicroscope with transillumination to obtain whole-mounts from the submucosa. Subsequently the whole-mounts were washed (2 x 10 min) with PBS plus detergent Triton X-100 0.5% (T) and incubated in blocking solution containing 2% bovine serum albumin (BSA) and 10% donkey serum (1 h). Then, the tissues were incubated for 48 h with primary anti-VIP antibody (Cat# T-4245.0400, AB_518686) diluted (1:500) in a mother solution (PBS, 1% BSA and 10% donkey serum), washed in PBS+T (2 x 10 min), incubated for 2 h with secondary antibody (Alexa Fluor 488; Cat# A21206, AB_10049650) diluted in mother solution (1:500) and washed with PBS (2 x 10 min). Subsequently the whole-mounts were mounted in slides with Prolong Gold Antifade Reagents. All of the procedures were performed at room temperature.

The slides were examined under a fluorescence microscope (Olympus FSX100). The VIP-immunoreactive neurons found in 40 microscopic fields under 20x objective were counted, with a total analyzed area of 5.87 mm^2^ /animal.

### Statistical analysis

The obtained data were subjected to the Kolmogorov-Smirnov normality test. Parametric data (body mass, food intake, blood glucose, intestinal length, blood biochemical analysis, intestinal enzyme activity, proliferative and serotoninergic cells number) were subjected to analysis of variance (ANOVA) followed by Tukey's post-test. For nonparametric data (morphometry of intestinal tunicas and quantification of VIP-immunoreactive neurons) the Kruskal-Wallis test followed by Dunns post-test was adopted. The results were presented as mean ± standard error (SEM). The statistical data were analyzed using GraphPad Prism program (GraphPad Software, version 5.0, USA) and considered significant at p<0.05.

## Results

### Physiological parameters

**Fig 1:**
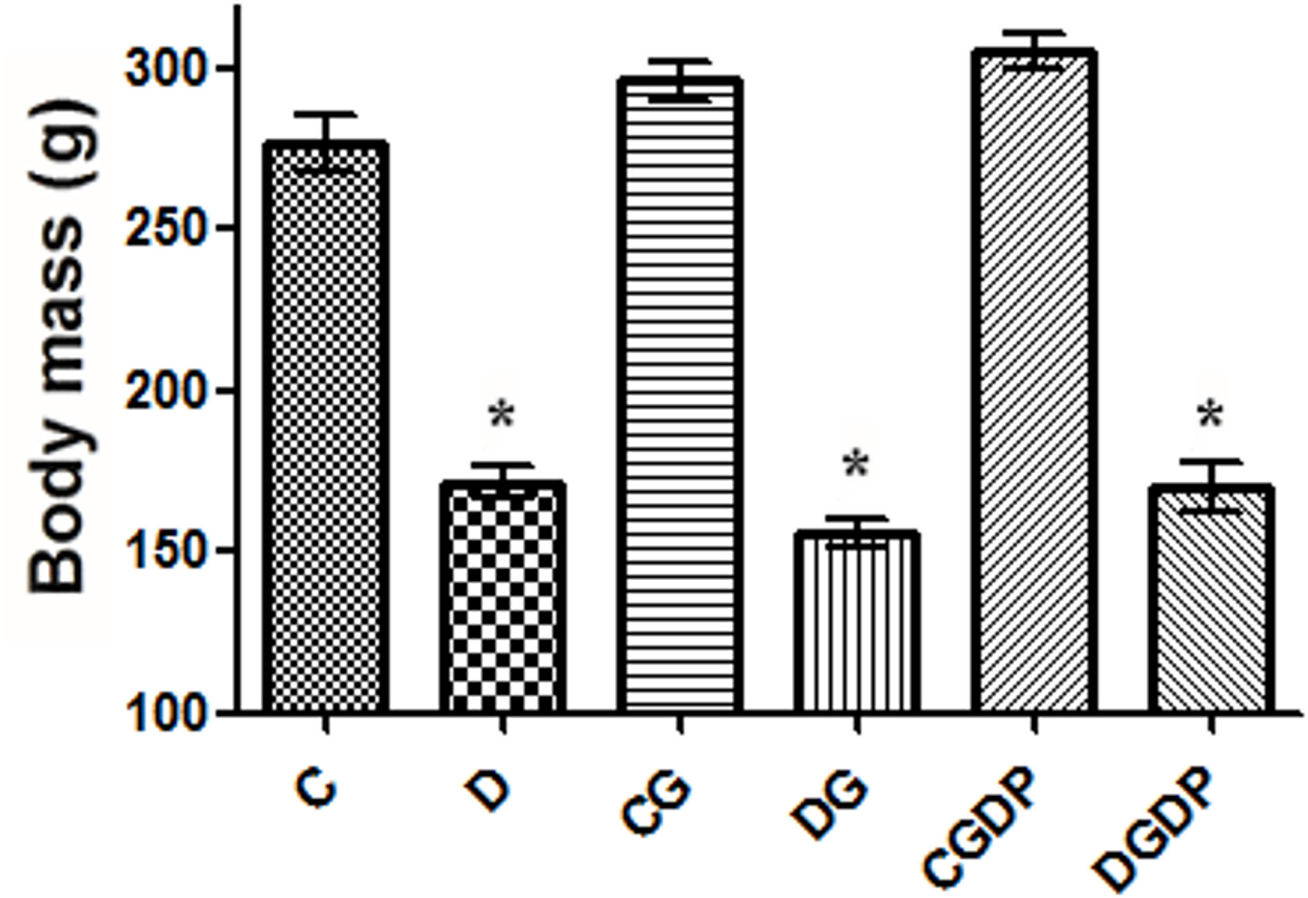
Body mass after 30 days receiving saline (C and D groups), L-glutamine (G) or glutamine dipeptide (GDP) in non diabetic rats (C groups), and diabetic rats (D). The animais were distributed in six groups: C, D, CG, DG, CGDP, and DGDP. Results presented as mean±SEM (n = 5/group). * p<0.05 compared to group C; one-way ANOVA and Tukey post test.

The streptozotocin-diabetic animals (D groups) showed the classic signs of the disease such as hyperglycemia (Table 1), lower body mass (Fig 1), and polyphagia (p<0.05) when compared to group C. The food intake was higher (p<0.05) in groups D (32.4±1.6 g/d) and DGDP (32.7±1.0 g/d) compared to the control group C (24.5±0.7 g/d), while that of the DG group was not significantly different (31.7±2.0 g/d). The CG (30.3±1.4 g/d) and CGDP (28.3±1.7 g/d) groups did not differ statistically from C (p>0.05). The supplementation with L-glutamine did not affect any of these parameters, but glutamine dipeptide (CGDP) promoted an increase (p<0.05) in body mass only relative to the control group C, while food intake and blood glucose levels (Table 1) were not affected (p>0.05). Also, polydipsia, polyuria, and constant diarrhea were observed in diabetic animals.

**Table 1:** Blood biochemical parameters after 30 days receiving saline (C and D groups), L-glutamine (G) or glutamine dipeptide (GDP) in non diabetic rats (C group), and diabetic rats (D). The animais were distributed in six groups: C, D, CG, DG, CGDP, and DGDP.

	C	D	CG	DG	CGDP	DGDP
**Fasting glucose (mg/dL)**	76.6±3.0	385.8±10.2*	67±2.5	417.0±31.3	82.0±2.9	394.6±20.7
**Fructosamine (mmol/L)**	0.88±0.02	1.55±0.08*	0.62±0.04	1.62±0.07	0.72±0.04	1.18±0.05#
**AST (U/L)**	41.15±2.7	205.40±36.1*	64.57±6.9	104.40±8.27	69.21±10.1	135.90±13.2
**ALT (U/L)**	12.08±1.3	232.30±33.7*	13.16±0.7	122.90±13.2#	21.80±1.8	260.30±14.3
**Triglycerides (mg/dL)**	28.50±1.2	71.38±7.8*	59.13±3.3	42.63±5.9#	58.70±11.0	71.83±10.2
**Total cholesterol (mg/dL)**	62.37±4.2	44.41±1.3	73.32±5.3	61.13±5.3#	86.35±1.3*	45.72±2.9
**HDL cholesterol (mg/dL)**	43.63±1.7	23.63±1.2*	38.63±1.5	38.13±3.8#	43.60±2.3	28.90±2.1
**Total protein (mg/dL)**	5.93±0.1	5.43±0.2	5.39±0.08	5.77±0.2	6.42±0.1	5.67±0.3
**Ammonia (mg/dL)**	3.05±0.06	3.42±0.1	3.05±0.08	3.236±0.2	2.89±0.1	2.73±0.2
**Urea (mg/dL)**	24.21±0.7	66.91±1.8*	30.69±1.5	58.97±1.8	30.86±2.7	66.87±3.2

Results expressed as mean±SEM (n = 5/group).

* p<0.05 vs. C group

# p<0.05 vs. D group

one-way ANOVA and Tukey post test.

Table 1 shows the blood biochemical profile of the animals. Confirming diabetes, high levels of fructosamine were detected (p<0.05) in D, DG and DGDP groups compared with their respective controls and a reduction (p<0.05) for DGDP compared to the untreated group (D). Liver enzymes (AST and ALT), indicative of liver tissue damage, showed high levels (p<0.05) in groups D and DGDP compared with their respective controls. There was a positive effect of the supplementation with L-glutamine in decreasing the transaminases (D group *vs*. DG group). On the other hand, L-glutamine or GDP supplementation did not affect transaminase levels (p>0.05) of control-treated groups (CG and CGDP).

Triacylglycerol increased (p<0.05) in D group compared with C group. Only L-glutamine avoided (p<0.05) the increase of blood triacylglycerol of D group. There was a slight, increase (p>0.05) of triacylglycerol in the CG and CGDP groups in comparison with CS group.

Total cholesterol increased (p<0.05) in group CGDP when compared to group C, whereas HDL cholesterol decreased (p<0.05) in group D. Supplementation with L-glutamine in the diabetic group (DG) maintained total cholesterol and HDL within the normality range for C group, while supplementation with the GDP did not recover this parameter in the DGDP group.

Total proteins and ammonia did not show changes (p>0.05) among the groups, while urea was increased (p<0.05) in all diabetic groups. But, no effect of supplementation was observed (Table 1).

### Intestinal morphophysiology

#### Intestinal enzyme activity

There was an increase (p<0.05) of alkaline phosphatase (AF) and lipase activity in D and DG groups (*vs*. C group). The DGDP group had a tendency of an increase (p>0.05) for these enzymes. The L-glutamine supplementation increased (p<0.05) the activities of β-galactosidase and maltase in DG group. Supplementation with GDP did not alter the enzymatic activities in both groups, except maltase (p<0.05) in diabetic animals DGDP group (Table 2).

**Table 2:** Enzymatic activity (U/g) of the jejunum after 30 days receiving saline (C and D groups), L-glutamine (G) or glutamine dipeptide (GDP) in non diabetic rats (C group), and diabetic rats (D). The animais were distributed in six groups: C, D, CG, DG, CGDP, and DGDP.

	C	D	CG	DG	CGDP	DGDP
**Alkaline Phosphatase**	0.056±0.007	0.091±0.004*	0.056±0.005	0.087±0.007	0.043±0.005	0.083±0.004
**β-galactosidase**	0.449±0.05	0.588±0.02	0.468±0.03	0.859±0.02#	0.447±0.03	0.719±0.05
**Lipase**	1.245±0.10	1.603±0,04*	1.126±0.07	1.592±0.11	0.947±0.08	1.313±0.08
**Maltase**	224.4±34.18	346.0±25.72	239.6±19.15	578.1±59.19#	324.7±11.31	515.3±28.39#

Results expressed as mean±SEM (n = 5/group).

* p<0.05 vs. C group

# p<0.05 vs. D group

one-way ANOVA and Tukey post test.

#### Intestinal morphometry

The intestinal morphology is presented in Table 3. D and DGDP groups had a greater length of the small intestine compared to C groups. A longer small intestine (p<0.05) was also observed in CG group.

**Table 3:** Small intestine length (cm) and jejunum morphometry (μm) after 30 days receiving saline (C and D groups), L-glutamine (G) or glutamine dipeptide (GDP) in non diabetic rats (C group), and diabetic rats (D). The animais were distributed in six groups: C, D, CG, DG, CGDP, and DGDP.

	C	D	CG	DG	CGDP	DGDP
**Small intestine lenght**	92.0±2.5	121.8±1.4*	104.8±1.8*	87.0±2.2	91.0±0.9	120.8±1.3
**Total wall**	742.9±4.5	790.2±3.0*	691.8±3.1*	774.8±4.5	786.3±3.2*	718.3±3.2#
**Mucosa**	642.6±3.9	693.8±2.7*	613.7±4.1*	697.8±4.5	668.0±3.1*	599.5±4.0#
**Submucosa**	23.1±0.2	18.2±0.1*	21.4±0.2*	17.6±0.1	27.0±0.2*	17.9±0.1
**Villus height**	460.4±2.6	463.1±1.9	407.9±2.3*	474.7±3.5	439.2±2.6*	405.1±2.7#
**Crypt depth**	177.1±1.1	191.7±1.0*	176.6±1.1	200.1±1.3#	191.7±0.9*	176.5±0.9#

Results expressed as mean±SEM (n = 5/group).

* p<0.05 vs. C group

# p<0.05 vs. D group

one-way ANOVA and Tukey post test.

The histological organization of the intestine was preserved in all groups. However, there were differences in the intestinal morphometric parameters between the groups.

It was observed that the jejunum of diabetic animals had total wall, mucosa and crypts depth larger than the controls, but a thinner submucosa (p<0.05). There was a positive effect (p<0.05) with glutamine dipeptide supplementation (DGDP) leading to reduction of the total wall, mucosa, crypt depth and villus height.

By contrast, non-diabetic animals showed a trophic effect (p<0.05) in total length, thickness of the mucosa and submucosa and crypt depth when supplemented with GDP. However, L-glutamine caused a reduction (p<0.05) of all the intestinal wall parameters, except the crypt depth in the CG group.

#### PCNA and serotonin immunohistochemistry

**Fig 2:**
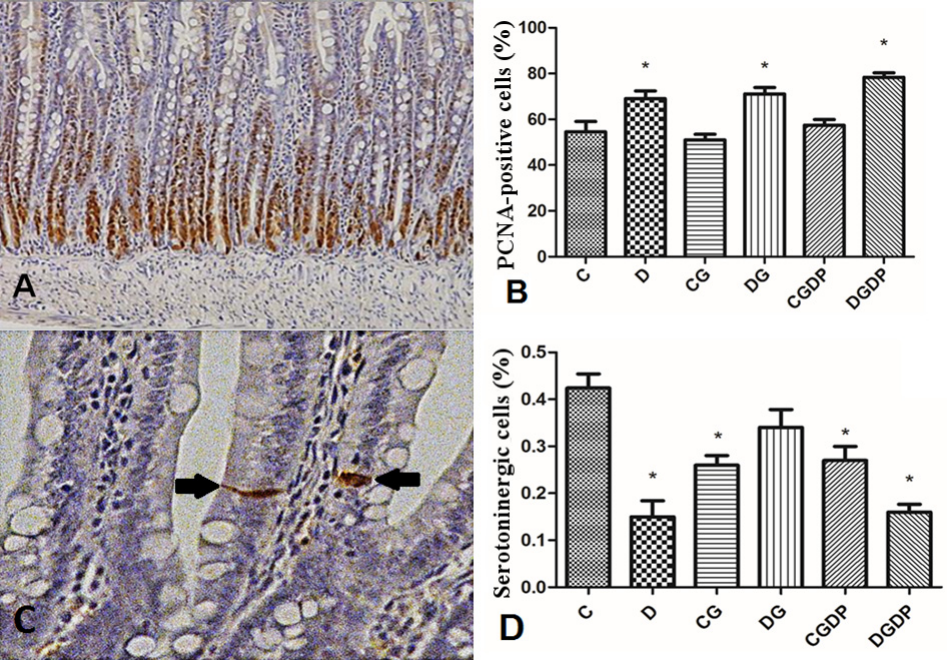
Immunostaining for PCNA and serotonin. (A) Jejunal mucosa of rats immunomarked for proliferation cell nuclear antigen (PCNA) (200x magnification); (B) Percentage of cells immunostained for PCNA after 30 days of experimentation; (C) Detail of enteroendocrine serotoninergic cells of jejunal mucosa (arrow) (200x magnification); (D) Percentage of immunostained cells for serotonin after 30 days receiving saline (C and D groups), L-glutamine (G) or glutamine dipeptide (GDP) in non diabetic rats (C group), and diabetic rats (D). The animais were distributed in six groups: C, D, CG, DG, CGDP, and DGDP. Results presented as mean±SEM (n = 5/group). * p<0.05 compared to group C; one-way ANOVA and Tukey post test.

Analysis of cell proliferation by PCNA labeling showed an increase of 26.6% (p<0.05) in the number of labeled cells in the crypts of D group vs. C group. L-glutamine supplementation showed no effect (p>0.05) on the CG and DG groups, as well as the C group supplemented with GDP (CGDP). There was an increase of 13.43% (p>0.05) in DGDP group relative to D (Fig 2B).

The immunostained serotoninergic cell index (Fig 2D) decreased by 64.62% (p<0.05) in group D compared with group C. L-glutamine (DG) prevented this reduction compared to the diabetic group (D), showing no difference (p>0.05) with the control group (C). The control group supplemented with GDP (CGDP) decreased by 36.32% (p<0.05) compared to C, while the diabetic supplemented group (DGDP) was not statistically different from group D.

#### Immunohistochemistry for submucous vipergic neurons

**Fig 3:**
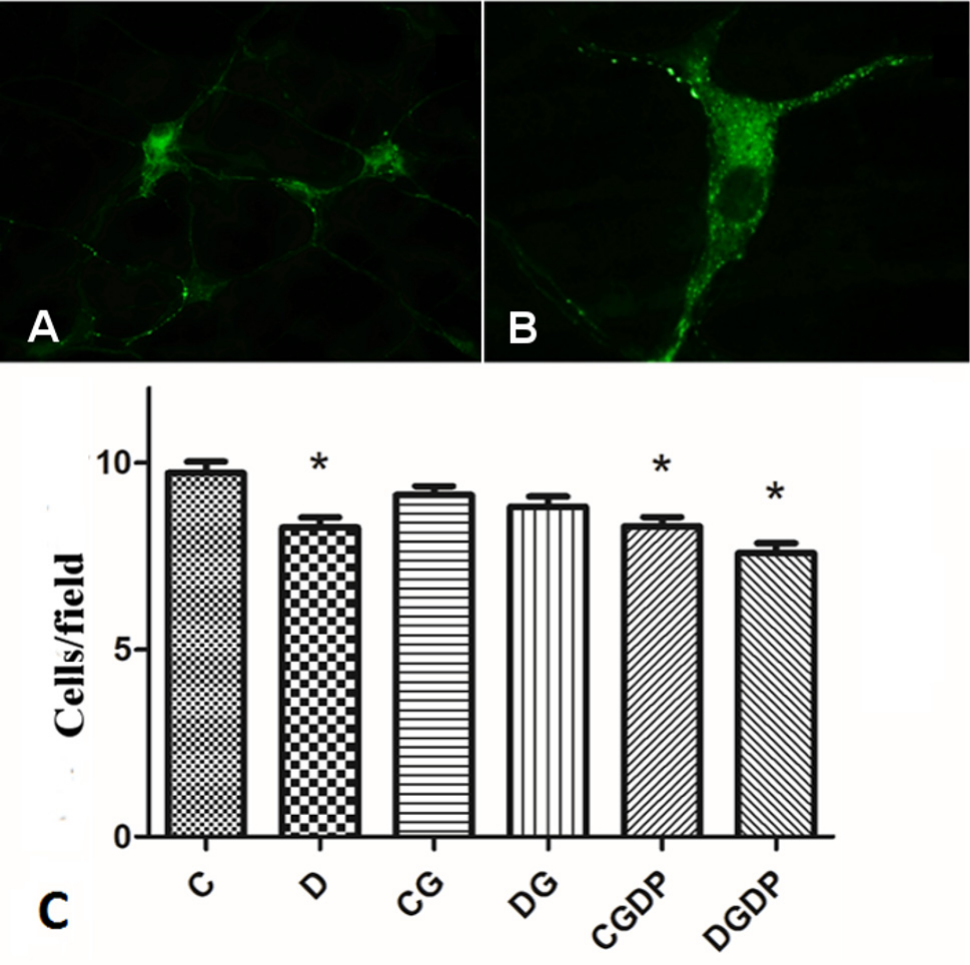
Submucous vipergic neurons. (A) Immunolabeling for vipergic ganglia of the jejunal submucous plexus of rats (200x magnification); (B) VIP-immunoreactive neuron (200x magnification); (C) Number of VIP-immunoreactive neurons in the jejunal submucous plexus of rats after 30 days receiving saline (C and D groups), L-glutamine (G) or glutamine dipeptide (GDP) in non diabetic rats (C group), and diabetic rats (D). The animais were distributed in six groups: C, D, CG, DG, CGDP, and DGDP. Mean values were obtained from the neuron count in 40 microscopic fields per animal, with a total area of 5.87 mm²/animal. Results presented as mean±SEM (n = 5/group). * p<0.05 compared to group C; Kruskal-Wallis test followed by Dunns post test.

The count of VIP-immunoreactive neurons (VIP-IR) (Fig 3) showed a 15.05% (p<0.05) reduction of these cells in D group (vs. C group). The L-glutamine supplementation promoted maintenance of the neuronal numbers in the DG group (vs. D group), keeping close to the number of neurons in C group, while it did not cause difference in the CG group (vs. C group). The CGDP group had a reduction (p<0.05) of 14.78% in the number of neurons i(vs. C group), while DGDP group showed no differences in comparison with D group.

## Discussion

### Physiological parameters

Experimental diabetes induced by streptozotocin was confirmed by the increased blood glucose and fructosamine (Table 1). The dose (60 mg/kg) was similar to those reported in the literature for rodents [18].

We observed not only hyperglycemia (Table 1) but also typical manifestations of the diabetic condition, such as hyperphagia, lower body mass [7, 19], polyuria, polydipsia, and diarrhea [20].

The L-glutamine amount of 248 mg/kg supplemented in this study was proportional to the specific amount of the amino acid in 400 mg/kg of glutamine dipeptide (GDP). The amount of GDP was similar to the maximum concentration used in humans that does not generate hyperammonemia [21].

Daily supplementation with L-glutamine by gavave during 30 days did not affect the physiopathological changes induced by streptozotocin (D group vs. DG group) and had no effect on the condition in the non-diabetic animals treated with L-glutamine (C group vs. CG group), as found by Tronchini *et al*. [19] and Tsai *et al*. [22]. Interestingly, in these studies [19, 22] they used the same period of supplementation but the animals received L-glutamine in the chow.

GDP behaves similarly to L-glutamine, i.e., in spite the fact that oral GDP did not show toxical effects [23], this dipeptide was not was able to improve the physiopathological changes induced by streptozotocin.

It is known that oral GDP performed better in terms of blood L-glutamine availability than oral L-glutamine [5]. This could be because the enterocytes utilize GDP at low rates [1, 2], which increases its availability for intestinal absorption. These differences between L-glutamine and GDP explain the fact that GDP is less effective for the gastrointestinal functions and more effective to functions that require plasma L-glutamine [5].

In agreement with the well-established increased protein catabolism due to the lack of insulin [24,25], streptozotocin-diabetic rats (D, DG and DGDP groups vs. C, CG and CGDP groups) showed high (P<0.05) blood levels of urea.

Lipid disorders in experimental diabetes are associated with insulin deficiency [26]. For example, insulin deficiency decreases the lipoprotein lipase activity increasing blood triacylglycerol [27]. In our study, triacylglycerol levels increased in D group, while the total cholesterol and HDL-cholesterol were reduced in D group if compared with C group. On the other hand, L-glutamine (DG group) increased HDL levels (DG group vs. D group). In agreement with these results, Badole *et al*. [28] reported hyperlipidemia and low HDL in a rat model of streptozotocin-induced type 2 diabetes plus nicotinamide; and an overall recovery in the lipid profile after treatment with L-glutamine.

The supplemented diabetic rats (DG group) showed an increased total cholesterol and HDL reaching the normal values observed in the non-diabetic rats (C group). However, this favorable effect of L-glutamine was not observed to GDP.

L-glutamine has beneficial effects in several diseases [29], but there are few studies about its supplementation in healthy individuals. Lowe *et al*. [30] showed that parenteral L-glutamine for seven days is well tolerated in healthy humans. In addition, in humans, Jian *et al*. [31] found no adverse effects after six days of supplementation with GDP. However, Schwimmer *et al*. [32] showed that L-glutamine is able to increase the absorption of triacylglycerol in the intestine of rats and this may be related to the elevation of blood lipids promoted by this amino acid [33]. Another possible justification for the effects of L-glutamine on the lipid profile could be the increased availability of energy substrates as consequence of the supplementation. However, GDP did not show these effects, in spite of its increased blood availability in comparison with L-glutamine [5].

Streptozotocin induces not only diabetes but also several additional physiopathological changes including hepatotoxicity [34–35]. However, this tissue damage was improved by supplementation with L-glutamine or GDP, as evidenced by the decreased levels of the enzymes AST and ALT (Table 1) in the DG or DGDP groups in comparison with D group. On the other side, the supplementation with L-glutamine or GDP, showed no significant effect on this enzymes in non-diabetic groups (C group vs. CG or CGDP groups).

Fructosamine, which reflects the average glycemic level over the preceding 2–3 weeks [36] was not influenced by L-glutamine. However, the supplementation with GDP was effective in reducing fructosamine levels.

### Intestinal morphophysiology

As previously demonstrated [37] we also observed increased intestinal enzymatic activity in diabetes, corroborating the literature, being related to greater proliferation and DNA expression in diabetic rats [38], which are directly related to higher absorption rates by the diabetic intestine [39]. The enzymes lipase and alkaline phosphatase are involved in lipid absorption [40]. The high lipid absorption in diabetic subjects contributed to the high levels seen in the lipid profile in diabetic animals. Since the supplemented control animals did not have these enzymes increased, L-glutamine could influence other pathways of lipid absorption.

The intestinal alkaline phosphatase is also related to the reduction of bowel inflammation by acting on the detoxification of bacterial lipopolysaccharides [40], and the increase observed in this study may also be a response caused by diabetic inflammation.

The disaccharidase increase (β-galactosidase and maltase) in diabetes is related to increased absorption of carbohydrates [37], resulting from intestinal hyperplasia [41], which was also demonstrated in our study, although not significantly. The supplementations used in this study did not prevent the changes of proliferation and intestinal expression caused by diabetes [38], consequently not affecting enzyme activity.

We verified higher length of the small intestine (SI) in the diabetic group compared to the control, supporting the literature concerning this parameter [18] and the weight of the SI, similarly reported by other authors [8]. These changes have a linear relationship with blood glucose [42], and may be caused by the influence of increased advanced glycation end-products in the gastrointestinal tract [43]. L-glutamine increased this parameter in the control group, while preventing the increase in the diabetic group. Being widely used as structural and energy molecule by the intestine, L-glutamine might have caused an increase in the length of the SI by greater availability of substrates, in addition to the effects on the increased proliferation and decreased cell death [44]. L-glutamine prevented hypertrophy of SI of diabetic rats (DG) during the experimental period, confirming its beneficial effects in stressful situations [45]. The GDP did not alter the intestinal length in CGDP or DGDP group compared to their controls, possibly due to the lower effect on the SI related to the differential absorption of dipeptides [5].

Despite the large mass loss due to catabolic state, the intestinal wall of the diabetic animal paradoxically increased in thickness, and this effect was mainly due to the increase in the mucosa [14]. This trophic effect of diabetes is related to hyperglycemia, since insulin therapy showed tissue normalization [46]. In the initial phase of diabetes, the enterocytes has increased proliferation and inhibition of apoptosis, which are responsible for the observed hyperplasia [47]. Corroborating the literature, diabetes increased the thickness of the jejunal wall in this study due to hyperplasia of the mucosa. Supplementation with L-glutamine was not able to mitigate the effects of diabetes on the intestinal wall. However, GDP was able to relieve the hyperplasia, which may be related to the reduction of AGE's, based on our fructosamine results.

The involvement of the intrinsic innervation is significant for the maintenance and adaptation of the intestinal structural change to ensure homeostasis of this organ. See *et al*. [48] reported that the myenteric plexus regulates mucosal cell proliferation in an inhibitory manner. Loss of myenteric neurons [49] and enhanced cell body area [50] of submucous neurons are reported in the literature, and can be associated with reduction of the total number of neurons in diabetes. Tronchini *et al*. [19] showed that L-glutamine had no effect on neuronal loss in the myenteric plexus of diabetic rats. The oxidative stress resulting from hyperglycemia [10] is the leading cause of neuronal death in diabetes, and could be affecting the intestinal wall, causing hyperplasia. As L-glutamine is rapidly metabolized in the mucosa, it would not be effective for the layers more distant from the intestinal lumen, such as the muscle tunicas where the myenteric neurons are located. On other hand, GDP could have more contact with this layer, influencing neurons to alleviate hyperplasia, as shown in this study, and reducing wall thickness.

The supplementation of L-glutamine to control animals (CG) reduced the jejunal wall thickness, in contrast with the increased length of SI evaluated in this study. This effect may be due to the increase in glutaminase enzyme activity in the intestine, which may occur in the control group supplemented with L-glutamine [51]. As the control group has no lack of L-glutamine, glutaminase could lead to a large production of L-glutamate, which is related to neurotoxicity [52]. The affected neurons could be unregulating proliferative and motor processes in the intestine. Tronchini *et al*. [19] and Hermes-Uliana *et al*. [53] also detected plastic and morphometric neuronal changes, respectively, by means of supplementation with L-glutamine. Tronchini *et al*. [19] observed a moderate reduction in height of the villi in control animals supplemented with L-glutamine, but in the ileum of rats, not the jejunum.

The reduction of the submucosa thickness in diabetic groups can be attributed to protein glycation in this tunica, especially collagen [54], affecting the mechanical properties of the gut, and hence its functions [8]. Despite being able to reduce blood fructosamine, GDP and L-glutamine as well were not able to prevent glycation in the submucosa. The supplementation for extended periods may result in better responses concerning this aspect.

Our data show that the increase of the mucosa in the diabetic animals results from increased crypt depth, since the height of the villi did not change. In the literature, increase of the villi and crypts is common in this disease [8]. This effect is more evident in the more distal parts of the intestine affected by diabetes [14], explaining the lower impact found in our villi data, together with the time of exposure to diabetes, of 30 days. The supplementation of diabetic rats with L-glutamine did not affect the villi height neither was able to reverse the hyperplasia of the crypts. Supplementation with GDP in diabetic rats (DGDP) reduced villi height and crypt depth as well the height of the mucosa, indicating a higher rate of apoptosis and/or cell migration compared to untreated diabetic groups. This effect can be attributed to an influence on the altered myenteric neurons in the diabetic condition which is associated with control of proliferation, as mentioned above [48]. This would be a positive influence of GDP on the myenteric plexus, buffering the neuronal changes observed.

The PCNA labeling demonstrates the increased cell proliferation in the crypts of diabetic animals, which confirms the increase of its depth. Miller *et al*. [38] also showed that the crypts proliferation in the small intestine is increased in diabetes. A higher mitotic index was found in the gut epithelium of denervated animals, showing connection between damage to the nervous tissue and mucosal cell proliferation [48]. However, the absence of change in height of the villi shows that there was no lower rate of apoptosis. This could indicate increased turnover, i.e., migration of intestinal wall cells. The two forms of L-glutamine supplementation did not normalize proliferation. L-glutamine has a certain ability to promote cell proliferation and migration [44], so maybe it was not able to normalize these factors, already high in the jejunum.

The supplementation with L-glutamine [11, 44] or GDP [55] has shown effectiveness in the model of intestine injuryby acetic acid and chemotherapy, through increased cell proliferation and/or reduced apoptosis of enterocytes, thus aiding in recovery. The lack of L-glutamine by intestinal tissue seems to be more effective in the presence of tissue injury [56].

The changes caused by diabetes in serotonin produced by the endocrine cells of the intestinal mucosa are contradictory in the literature. Takahara *et al*. [57] detected increase in serotonin levels in the SI of diabetic rats induced by streptozotocin. By contrast, there are also reports of reduction in these levels in SI of diabetic animals induced by streptozotocin [58] and alloxan [59]. Our results show reduction of serotoninergic cell numbers in the jejunum in diabetic rats. Gastrointestinal problems resulting from diabetes may also be related to this reduction, since serotonine has a role in the regulation of peristalsis, influencing the enteric neurons [60]. These neurons also have the activity of its serotoninergic receptors altered in diabetes [57]. Cicin-Sain and Jernej [59] point out that this reduction is due to lower production of serotonin, caused by the reduction of its substrates/precursors in diabetes. It is also possible that this is an organ response to neuropathy, to offset the high motor activity caused by diabetes neuronal deregulation [61].

In our study, L-glutamine prevented the decrease of serotonininergic cell numbers, while GDP did not change this parameter, probably due to the increased availability of the free form to the intestine. The literature lacks information associating L-glutamine and serotonin in the gut of diabetic rats.

Our results show that the installation and maintenance of a diabetic profile for 30 days reduces the number of jejunal submucous plexus vipergic neurons. Adeghate *et al*. [62] obtained reduced numbers of vipergic neurons in the gastroduodenal region of diabetic rats after four weeks, while Hermes-Uliana *et al*. [53] detected increase of this subpopulation in the jejunum of diabetic rats after 120 days, which was considered as an adaptive response to promote neuronal protection. The imbalance of neurotransmitters caused by diabetic neuropathy is one of the causes of the typical gastrointestinal diabetic symptoms. This neuropathy is mainly a consequence of oxidative stress [9, 10], caused by hyperglycemia. Diabetic neuropathy, with reduction of cell body area and loss of neurons, has already been described in the myenteric [63] and submucous [13] plexuses.

There may also be a relationship between vipergic neuronal change and the reduced amount of serotonininergic cells discussed above. As part of the lost neuronal inhibitory function, there would be lower production of serotonin, a motility promoter, in response to the faster intestinal transit in an attempt to counterbalance the motor function.

The neuroprotection caused by L-glutamine in the diabetic group (DG) in this study has been reported in the literature for vipergic [12, 53] and general [64] populations. Supplementation with L-glutamine has proven effective in minimize oxidative stress [22, 28, 65, 66], increasing its availability to tissues under stress [67]. The GDP also has antioxidant potential [65], but its supplementation was not able to protect the submucous neurons. This effect was attributed to the low effect of GDP on enterocytes.

## Conclusions

The supplementation with L-glutamine or GDP did not affect the typical physiopathological changes induced by streptozotocin administration. However, L-glutamine produced positive better responses than GDP on lipid fractions and transaminases levels. Moreover, with respect to jejunum morphophysiology in diabetic rats, it was preserved the number of enteroendocrine serotoninergic cells and vipergic neuronal subpopulation of the submucous plexus. On the other hand, the supplementation with GDP was effective in reducing fructosamine levels and intestinal mucosa hyperplasia in diabetic rats. Thus, the results open the possibility of using mixed supplementation (L-glutamine plus GDP) to improve the beneficial effects of each substance.

## Supporting Information

S1 Dataset Excel spreadsheets containing, in separate archives, the underlying numerical data for Tables 1, 2, 3, and Figs 1, 2 and 3. (RAR)
